# Ocular A-to-I RNA editing signatures associated with SARS-CoV-2 infection

**DOI:** 10.1186/s12864-024-10324-z

**Published:** 2024-05-01

**Authors:** Yun-Yun Jin, Ya-Ping Liang, Wen-Hao Huang, Liang Guo, Li-Li Cheng, Tian-Tian Ran, Jin-Ping Yao, Lin Zhu, Jian-Huan Chen

**Affiliations:** 1 Laboratory of Genomic and Precision Medicine, Wuxi School of Medicine, Jiangnan University, Wuxi, Jiangsu China; 2 Joint Primate Research Center for Chronic Diseases, Institute of Zoology of Guangdong Academy of Science, Jiangnan University, Wuxi, Jiangsu China; 3 Jiangnan University Brain Institute, Wuxi, Jiangsu China; 4 Jiangnan University-Xinshijie Eye Hospital Joint Ophthalmic Research Center, Wuxi, Jiangsu China

**Keywords:** A-to-I RNA editing, SARS-CoV-2, Ocular surface, Retina, Epitranscriptomic

## Abstract

Ophthalmic manifestations have recently been observed in acute and post-acute complications of COVID-19 caused by SARS-CoV-2 infection. Our precious study has shown that host RNA editing is linked to RNA viral infection, yet ocular adenosine to inosine (A-to-I) RNA editing during SARS-CoV-2 infection remains uninvestigated in COVID-19. Herein we used an epitranscriptomic pipeline to analyze 37 samples and investigate A-to-I editing associated with SARS-CoV-2 infection, in five ocular tissue types including the conjunctiva, limbus, cornea, sclera, and retinal organoids. Our results revealed dramatically altered A-to-I RNA editing across the five ocular tissues. Notably, the transcriptome-wide average level of RNA editing was increased in the cornea but generally decreased in the other four ocular tissues. Functional enrichment analysis showed that differential RNA editing (DRE) was mainly in genes related to ubiquitin-dependent protein catabolic process, transcriptional regulation, and RNA splicing. In addition to tissue-specific RNA editing found in each tissue, common RNA editing was observed across different tissues, especially in the innate antiviral immune gene *MAVS* and the E3 ubiquitin-protein ligase *MDM2*. Analysis in retinal organoids further revealed highly dynamic RNA editing alterations over time during SARS-CoV-2 infection. Our study thus suggested the potential role played by RNA editing in ophthalmic manifestations of COVID-19, and highlighted its potential transcriptome impact, especially on innate immunity.

**Supplementary Information:**

The online version contains supplementary material available at 10.1186/s12864-024-10324-z.

## Introduction

Since 2019, the world has been facing a pandemic of coronavirus disease 2019 (COVID-19) caused by the SARS-CoV-2 virus, resulting in significant impacts on global healthcare and economies [1]. Symptoms of COVID-19 vary from being asymptomatic to severe [2, 3]. SARS-CoV-2 infection could cause viral pneumonia [4], but can also affect the heart, liver, kidney, brain [5–9], and eyes [10]. Understanding such infections in other tissues and organs than the lung is essential for the control and treatment of acute and post-acute COVID-19 sequelae.

The ocular surface is an area directly exposed to the air and thus is vulnerable to possible viral infection. SARS-CoV-2 RNA can be detected in the ocular surface [11], and its viral particles and RNA are detected in different layers of the retina [12, 13]. Additionally, the interaction between spike protein and angiotensin-converting enzyme 2 (*ACE2*) mediates SARS-CoV-2 entry into the human cells, and transmembrane serine protease 2 (*TMPRSS2*) is responsible for the initiation of spike protein and promotes such an interaction [14–17]. ACE2, TMPRSS2, and other accessory entry factors are expressed in the conjunctiva, limbus, cornea, sclera, and retinal organoids [18–20]. Ocular manifestations of COVID-19 include conjunctivitis, keratitis, uveitis, retinitis, etc. [10, 21–35]. However, the underlying mechanisms related to ocular symptoms in COVID-19 remain to be further investigated.

Adenosine-to-inosine (A-to-I) RNA editing mediated by adenosine deaminases that act on RNA (*ADARs*) was the most common canonical RNA editing in mammals [36]. Three members of ADARs are encoded in the human genome, including *ADAR*, *ADARB1*, and *ADARB2*. *ADAR* (also known as *ADAR1*) and *ADARB1* (also known as *ADAR2*) are expressed in many tissues and demonstrate catalytic activity for adenosine deamination, whereas *ADARB2* (also known as *ADAR3*) is mainly present in the brain and no adenosine deaminase activity has been reported [37–39]. As an important component of epigenetics, RNA editing plays an important role in various physiological and pathological processes [40]. It is associated with the pathogenesis of neurodegenerative diseases such as amyotrophic lateral sclerosis (ALS), Parkinson's disease, and Alzheimer's disease [41]. In addition, A-to-I RNA editing is an important component of innate and adaptive immunity and plays an important role in the host’s antiviral responses [38], such as those to the Ebola virus, hepatitis virus, SARS, and SARS-CoV-2 [42, 43]. Meanwhile, ADARs influenced SARS-CoV-2 infection in vivo [44]. Moreover, our recent study revealed a possible link between host RNA editing and infection with single-strand RNA viruses, including SARS-CoV-2 in mouse models [45]. However, ocular A-to-I RNA editing during SARS-CoV-2 infection remains uninvestigated in COVID-19 patients.

Herein, we performed a transcriptome-wide analysis to examine the RNA editing profiles of SARS-CoV-2 infections in five ocular tissues, to identify SARS-CoV-2 infection-associated signatures of host RNA editing across tissues. Our findings highlight both the similarities and differences in host RNA editing during SARS-CoV-2 infections and provide valuable insights into the epigenetic mechanisms of RNA editing underlying the ophthalmic manifestations of SARS-CoV-2 infection.

## Materials and methods

### RNA-Seq dataset downloads

We downloaded three RNA-Seq datasets containing 37 samples of five ocular tissues from the European Nucleotide Archive (ENA) (https://www.ebi.ac.uk/ena). These datasets include PRJNA790648 [46], PRJNA688734 [47], and PRJNA731890 [48]. Dataset PRJNA688734 contains samples from the cornea, limbus, and sclera isolated from human donor tissues and passaged in tissue culture. Cells of the three tissue were infected with SARS-CoV-2 in triplicate and their RNA was collected at 24 h post-infection (hpi), and compared to mock samples (*N* = 3 each). The PRJNA790648 contains mock (*N* = 4) and SARS-CoV-2 infected (*N* = 3) ex vivo cultures of an air–liquid interface organotypic conjunctival epithelial model epithelia infected. Dataset PRJNA731890 [48] contained samlpes of human stem cell-derived retinal organoids that were mock or infected with SARS-CoV-2 collected at 24 and 96 hpi (*N* = 3 each). Details of these datasets are shown in Table 1.

**Table 1:** Details of the datasets analyzed in the current study

Bio Project Accession	Tissue	Mock (*N*=18)	Infected (*N*=19)	Viral strain	Multiplicity of infection (MOI)	Incubated time (h)	Contributors	Citation
PRJNA790648	conjunctiva	3	4	BetaCoV/England/2/2020	0.5	24	Jackson, et al., 2022	[46]
PRJNA688734	cornea	3	3	SARS-CoV-2/USA-WA1/2020	1.0	24		
	limbus	3	3				Eriksen, et al., 2021	[47]
	sclera	3	3					
PRJNA731890	retinal organoids (24h)	3	3		0.01	24		
	retinal organoids (96h)	3	3	hCoV19/Germany/FI1103201/2020		96	Menuchin-Lasowski, et al., 2022	[48]

### Read alignment

The raw sequencing reads were processed using a workflow as previously described [49]. In summary, raw sequencing data quality was assessed using FASTQC [50]. Read alignment was performed using RNA STAR (version 2.7.0e) and the human genome reference sequence (UCSC hg38) [50]. Base quality scores were then recalibrated using GATK (version 4.1.3) [51] after filtering duplicated reads using SAMtools (version 1.9) [52].

### RNA editing identification

VarScan (version 2.4.4) and the Ensembl Variant Effect Predictor (VEP) were used for the identification and annotation of A > G single nucleotide variations (SNVs) [53, 54]. SNVs meeting specific criteria were selected, including a base quality of ≥ twenty-five, total sequencing depth of ≥ ten, alternative allele depth of ≥ two, and alternative allele frequency (AAF) of ≥ 1%. SNVs found in the REDIportal V2.0 database were considered high-confidence A-to-I RNA editing sites [55]. Additional filtering criteria were applied to the remaining SNVs, such as excluding those located in homopolymer runs (≥ five nucleotides) or simple repeats, mitochondrial genes, within six nucleotides from splice junctions, within one nucleotide from RNA insertion-deletion (INDEL), within 4% of the ends of reads, annotated as known variants in the dbSNP database Build 142, and those with AAF values of 100% or between 40 and 60% in more than 90% of NC and infected samples. High-confidence A-to-I RNA editing sites were retained for subsequent data analysis, defined as those with editing levels ≥ 1% and observed in two or more samples.

### Functional enrichment analysis

Gene Ontology (GO) and Kyoko Encyclopedia of Genes and Genomes (KEGG) pathway analyses were conducted using online prediction tools, including DAVID (https://david.ncifcrf.gov/tools.jsp), Enrichr (https://maayanlab.cloud/Enrichr/), and an online tool (http://www.bioinformatics.com.cn/) [56]. Significance was determined based on a false discovery rate (FDR) < 0.05.

### RNA binding protein (RBP) binding site prediction

To gain deeper insights into the potential functional consequences of RNA editing, RBPmap (http://rbpmap.technion.ac.il) was used to predict RNA binding protein sites that coincided with RNA editing sites [57].

### Statistical analysis

The general linear model (GLM) and likelihood ratio test were used to compare RNA editing levels between NC and SARS-CoV-2 infected tissues and identify differential RNA editing (DRE), which could be associated with SARS-CoV-2 infection. Empirical *P*-values (*P*_GLM_) were calculated using the likelihood ratio test. For sites with *P*_GLM_ less than 0.05, *Fisher’s* exact test was further used to compare the sequencing depth of the reference and alternative alleles in both groups. We applied the Benjamini–Hochberg method to calculate the false discovery rate (FDR). RNA editing sites with FDR less than 0.05 were considered differentially edited. Additionally, we analyzed the correlation between RNA editing and gene expression levels using *Spearman’s* correlation.

## Results

### A-to-I RNA editing profiles in ocular tissues

**Fig. 1:**
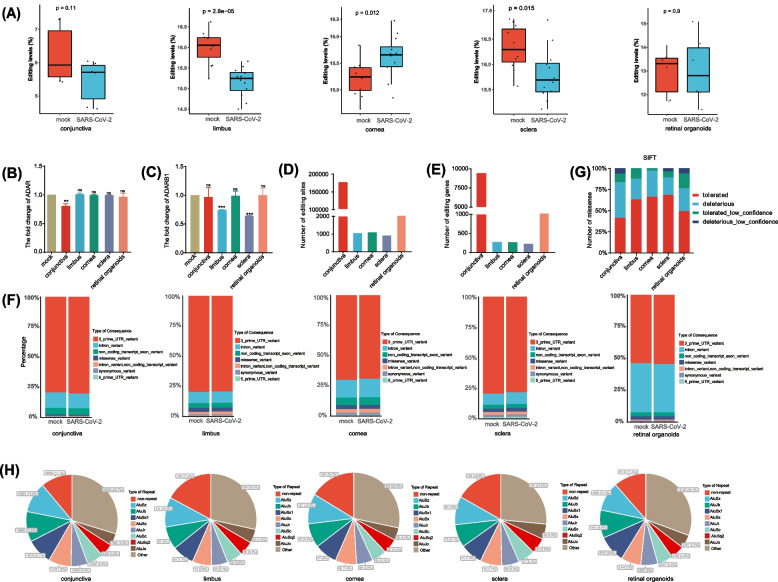
A-to-I RNA editing was identified from transcriptomes of the five ocular tissues in the current study. **A** The average A-to-I RNA editing level of the conjunctiva, limbus, cornea, sclera, and retinal organoids between mock and SARS-CoV-2 infection. **B**-**C** The fold change in the expression level of *ADAR* and *ADARB1* genes, (**D**-**E**) the number of A-to-I RNA editing sites and genes, and (**F**) the functional types of variants resulting from A-to-I RNA editing in mock and SARS-CoV-2 infection. **G** SIFT prediction of the missense RNA editing variants. **H** RNA editing sites distribution in repetitive elements. A two-tailed unpaired Student’s t-test was used to analyze the significance; *P < 0.05; **P < 0.01; ***P < 0.001; ns, no significance

To assess whether and how ocular A-to-I RNA editing was involved during SARS-CoV-2 infection, we searched and downloaded the publicly available RNA-seq datasets containing ocular tissues or organoids infected with SARS-CoV-2. The results showed the average RNA editing level was decreased in the conjunctiva, limbus, and sclera, but increased in the cornea, with no significant changes between mock and infected retinal organoids (Fig. 1A). Meanwhile, upon infection, *ADAR* expression decreased in the conjunctiva, whereas *ADARB1* expression decreased in the limbus and sclera (Fig. 1B, C). Furthermore, a total of 178558 editing sites were identified in the conjunctiva, 1084 in the limbus, 1140 in the cornea, 941 in the sclera, and 44716 in retinal organoids (Fig. 1D). For the editing genes, 9520, 290, 292, 246, and 3622 were found in conjunctiva, limbus, cornea, sclera, and retinal organoids, respectively (Fig. 1E). Most of these RNA editing sites were in the introns and 3’-untranslated region (UTR) (Fig. 1F). Although missense variants only accounted for a small fraction, they could affect protein structure and stability [58]. So sorts intolerant from tolerant (SIFT) predicted 461 (48.6%), 8 (23.5%), 11 (30.6%), 7 (24.1%), and 134 (32.9%) of the missense variants to be possibly deleterious in the conjunctiva, limbus, cornea, sclera, and retinal organoids, respectively (Fig. 1G). More than half of the repeat sequences of these editing sites were located in the *Alu* repeat elements (Fig. 1H). These results pointed to distinct alterations of RNA editing profiles during SARS-CoV-2 infections.

### Comparison of A-to-I RNA editing profiles of SARS-CoV-2 infected ocular tissues

**Fig. 2:**
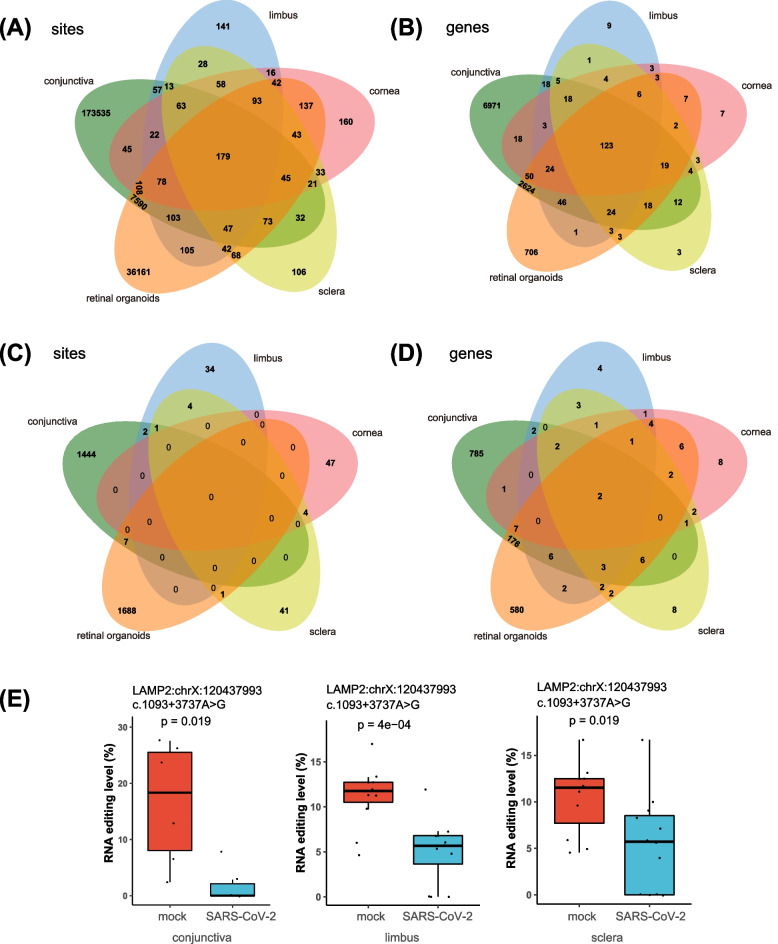
The similarities and differences of A-to-I RNA editing between the five ocular tissues. **A**-**B** Venn plot showing total A-to-I RNA editing sites (**A**) and genes (**B**) shared by the five ocular tissues. **C**-**D** Venn plot showing differential A-to-I RNA editing sites (**C**) and genes (**D**) shared by five ocular tissues. **E** The editing levels of *LAMP2* (chrX:120,437,993), a common DRE site associated with COVID-19 in the conjunctiva, limbus, and sclera

We then looked into the RNA editing profile of each tissue. The five tissue types shared 179 editing sites, and 173,535, 141, 160, 106, and 36,161 specific sites were uniquely found in the conjunctiva, limbus, cornea, sclera, and retinal organoids, respectively (Fig. 2A). For edited genes, 123 were shared by the five tissue types, whereas 6971, 9, 7, 3, and 706 were uniquely observed in the conjunctiva, limbus, cornea, sclera, and retinal organoids, respectively (Fig. 2B). Further comparison of DRE sites and genes among the five tissue types showed 1444, 34, 47, 41, and 1688 tissue-specific DRE sites in the conjunctiva, limbus, cornea, sclera, and retinal organoids, respectively (Fig. 2C), and 785, 4, 8, 8, and 580 tissue-specific genes were found in the conjunctiva, limbus, cornea, sclera, and retinal organoids, respectively (Fig. 2D). Notably, the five tissue types shared differential RNA editing in *MAVS* and *MDM2* (Table 2), whereas *LAMP2* (chrX:120,437,993) RNA editing was differentially edited across the conjunctiva, limbus, and sclera (Fig. 2E).

**Table 2:**
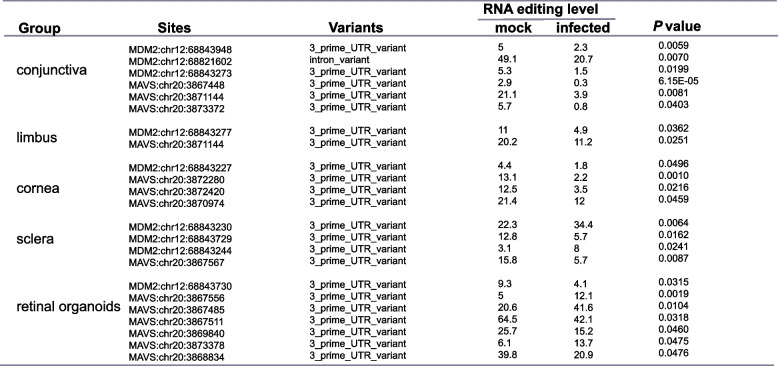
DRE sites in *MDM2* and *MAVS*

### Functional relevance of *SARS-CoV-2 infection-ass*ociated A-to-I RNA editing in ocular tissues

**Fig. 3:**
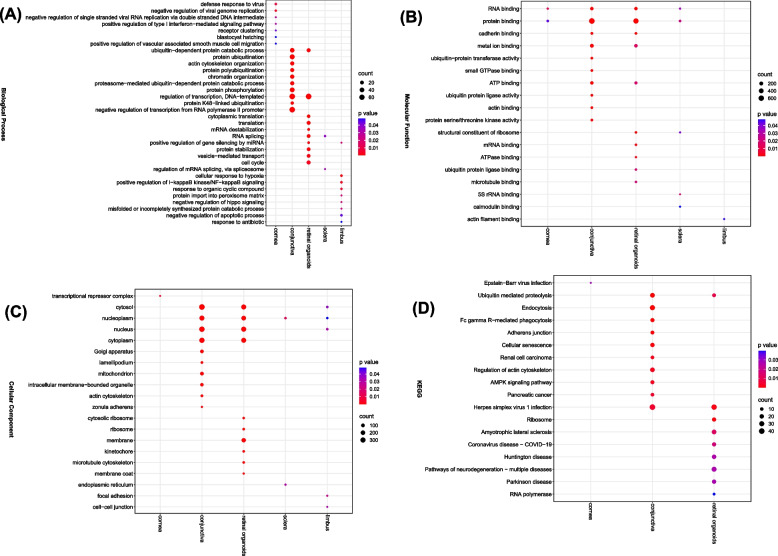
Functional enrichment analysis of SARS-CoV-2 infection-associated A-to-I RNA editing in the five ocular tissues. **A**-**D** The enrichment analysis results of biological processes (**A**), molecular functions (**B**), cellular components (**C**), and KEGG pathway (**D**) enriched by genes with DRE are shown

Functional enrichment analysis using these DRE genes was then used to understand the impact of A-to-I RNA editing changes on biological functions during SARS-CoV-2 infection. Notably, the results in Fig. 3 showed more evident functional enrichment of DRE in the conjunctiva and retinal organoids than in the other three tissues included in the current study. The common enrichment in DRE genes consisted of biological processes mainly related to ubiquitin-dependent protein catabolic process, regulation of transcription, RNA splicing, and positive regulation of gene silencing by miRNA (Fig. 3A); common molecular functions mainly related to RNA binding, protein binding, cadherin binding, and metal ion binding (Fig. 3B); common cellular components included the cytosol, nucleoplasm, nucleus, and cytoplasm (Fig. 3C), and common KEGG pathways were mainly related to ubiquitin-mediated proteolysis and herpes simplex virus 1 infection (Fig. 3D). Tissue type also had its unique enriched features, especially those related to viral infection, such as biological processes related to defense response to virus, negative regulation of viral genome replication, and positive regulation of type I interferon-mediated signaling pathway were uniquely found in the cornea, (Fig. 3A), and KEGG pathways of coronavirus disease-COVID-19 were unique to the retinal organoids (Fig. 3D).

### SARS-CoV-2 infection-associated A-to-I RNA editing in ocular surface tissues

**Fig. 4:**
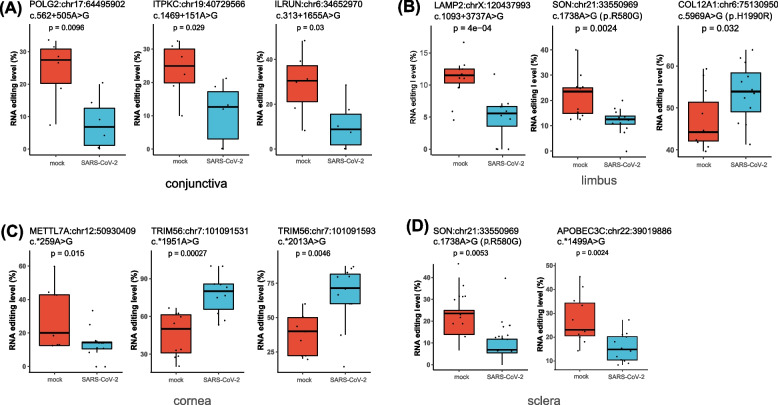
SARS-CoV-2 infection-associated A-to-I RNA editing in ocular surface tissues. The main DRE sites (no more than 50, ranked by *P*-values) in the conjunctiva (**A**), limbus (**B**), cornea (**C**), and sclera (**D**) are shown

We then focused on the role of A-to-I RNA editing during this process. 1454 DRE sites in 991 genes, 41 DRE sites in 33 genes, 51 DRE sites in 38 genes, and 51 DRE sites in 35 genes were identified in the conjunctiva, limbus, cornea, and sclera infected (Fig. S1). Notably, the top 50 DRE sites (ranked by empirical *P*-values) in the different tissue types (Fig. S2) were strongly correlated with *ADAR* and *ADARB1* expression (Table 3), pointing to an active role of ADARs during ocular infection of SARS-CoV-2. In conjunctiva, the RNA editing levels of DNA polymerase gamma 2 (*POLG2*:chr17:64,495,902), inositol-trisphosphate 3-kinase C (*ITPKC*:chr19:40,729,566), and inflammation and lipid regulator with UBA-like and NBR1-like domains (*ILRUN*:chr6:34,652,970) decreased significantly after SARS-CoV-2 infection (Fig. 4A). In the limbus, the editing level of lysosomal associated membrane protein 2 (*LAMP2*:chrX:120,437,993), SON DNA and RNA binding protein (*SON*:chr21:33,550,969) deceased, and that of collagen type XII alpha 1 chain (*COL12A1*:chr6:75,130,950) increased (Fig. 4B). In the cornea, the editing level of methyltransferase like 7A (*METTL7A*:chr12:5,093,040) deceased after SARS-CoV-2 infection and tripartite motif containing 56 (*TRIM56*:chr7:101,091,531 and chr1:101,091,593) increased after SARS-CoV-2 infection (Fig. 4C). Additionally, both *METTL7A* and *TRIM56* expressions significantly increased after SARS-CoV-2 infection. The editing level and mRNA expression level of *METTL7A* showed a negative correlation, whereas *TRIM56* showed a positive correlation (Fig. S3A-S3D). *SON* (chr21:33,550,969) also showed a decreased editing level in the limbus and sclera upon infection (Fig. 4B, D). Among these sites in the sclera, the most important is that apolipoprotein B mRNA editing enzyme catalytic subunit 3C (*APOBEC3C*), a member of the cytidine deaminase gene family related to C-to-U RNA editing, showed a decreased editing level (Fig. 4D), which was negatively correlated its up-regulated gene expression (Fig. S3E).

**Table 3:**
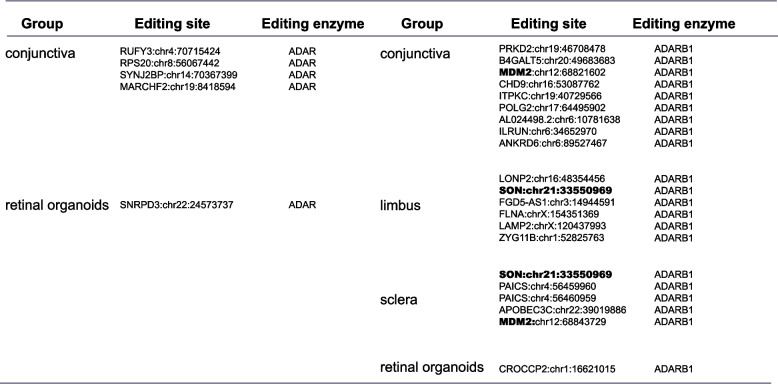
Sites correlated with *ADAR* and *ADARB1*

### Temporal dynamics of A-to-I RNA editing in the retinal organoids during SARS-CoV-2 infection

**Fig. 5:**
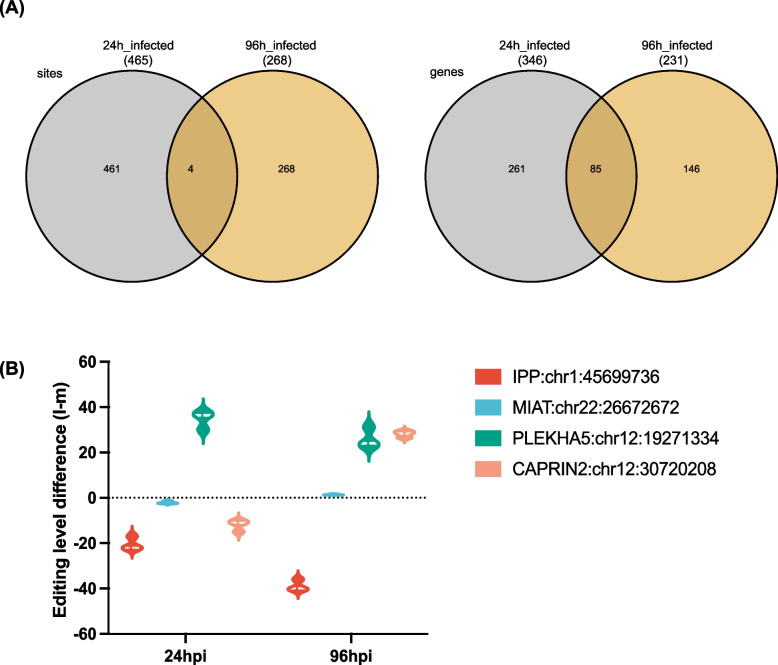
Temporal dynamics of SARS-CoV-2 infection-associated A-to-I RNA editing in the retinal organoids. **A** 4 sites and 85 genes were shared by 24 hpi and 96 hpi groups, respectively. **B** The editing level difference of four shared sites between 24 and 96 hpi groups

A total of 1696 DRE sites in 799 DRE genes were identified in the retinal organoids transcriptome upon SARS-CoV-2 infection (Fig. S1). The retinal organoids infected were divided into 24 hpi and 96 hpi groups to investigate the possible role of A-to-I RNA editing during infection. More DRE sites were found at 24 hpi than 96 hpi, with 465 and 272 DRE sites found in 346 and 231 DRE genes at 24 hpi and 96 hpi, respectively (Fig. 5A). These DRE sites varied a lot from 24 to 96 hpi, with only 4 sites and 85 genes shared by 24 hpi and 96 hpi (Fig. 5A). The genes with four common DRE sites included intracisternal A particle-promoted polypeptide (*IPP*:chr1:45,699,736), myocardial infarction associated transcript (*MIAT*:chr22:26,672,672), pleckstrin homology domain containing A5 (*PLEKHA5*:chr12:19,271,334), and caprin family member 2 (*CAPRIN2*:chr12:30,720,208). The editing level of *IPP* and *PLEKHA5* decreased both at 24 hpi and 96 hpi (Fig. 5B). In contrast, the editing levels of *MIAT* and *CAPRIN2* increased both at 24 hpi and 96 hpi compared to mock retinal organoids (Fig. 5B).

## Discussion

The COVID-19 pandemic caused by SARS-CoV-2 has affected hundreds of millions worldwide (https://covid19.who.int/). In addition to respiratory symptoms, ocular manifestations are also reported in COVID-19 [10, 21–35]. By conducting a comprehensive epitranscriptomic analysis of ocular tissues, our current study provided evidence supporting the potential role of A-to-I RNA editing in ocular manifestations during SARS-CoV-2 infection.

Our study analyzed RNA sequencing data from publicly available ocular tissue datasets, including the conjunctiva, limbus, cornea, sclera, and retinal organoids, to determine the potential role of A-to-I RNA editing in SARS-CoV-2 infection-related ocular diseases. The results showed significant changes in RNA editing profiles in various ocular tissues after COVID-19 infection. Specifically, the limbus, cornea, and sclera showed fewer RNA editing sites and genes when compared to the conjunctiva and retinal organoids (Fig. 1D, E), possibly due to the lower sequencing depth of the data in the tissues. Although emerging studies have reported ocular symptoms in COVID-19 patients, such as conjunctival congestion, blurred vision, and foreign body sensation [24, 59], it remains unclear how these symptoms are caused by SARS-CoV-2. Our results found that infection with SARS-CoV-2 in ocular tissues substantially altered RNA editing, suggesting a potential role of RNA editing in the ocular infection of SARS-CoV-2 and its ocular manifestations.

Most of these ocular RNA editing sites were within *Alu* repetitive elements (Fig. 1H). *Alu* elements are abundant short interspersed nuclear elements in the human genome and have been shown to play a critical role in regulating gene expression and alternative splicing [60]. In addition, *Alu* elements are also involved in RNA editing as they were hot spots recognized by ADAR enzymes. Our findings suggested that these repetitive elements may play a key role in RNA editing, which contributes to the relationship between RNA editing and ocular SARS-CoV-2 infection.

Common DRE sites were observed in two or more tissues of the conjunctiva, limbus, cornea, sclera, and retinal organoids (Fig. 2C, E). For example, DRE in *LAMP2* was shared among the conjunctiva, limbus, and sclera might suggest the involvement of lysosome-related functions in these tissues during SARS-CoV-2 infection. Importantly, our study also highlighted *MAVS* and *MDM2* genes with common DRE sites across different ocular SARS-CoV-2 infections, pointing to common regulatory mechanisms in these tissues during the infection. It has been confirmed that SARS-CoV-2 is involved in the intrinsic antiviral response mediated by MAVS [61], and the inhibition of MDM2 might protect the eye from SARS-CoV-2 infections [62].

Our study also found RNA editing sites significantly correlated with gene expression, suggesting possible *cis*-regulating of gene expression in ocular tissues after SARS-CoV-2 infection. In addition, DRE varied among ocular tissues, particularly the retinal organoids, and conjunctiva, in which SARS-CoV-2 infections might affect the visual and immune functions [63, 64]. In addition, each tissue type had its unique DRE genes, especially those related to viral infections, suggesting a critical yet divergent role of A-to-I RNA editing in the immune response to ocular viral infections due to functional differences among conjunctiva, cornea, and retinal organoids (Fig. 3D).

In addition, we also found that *APOBEC3C* (Fig. 4D), a catalytic subunit of lipoprotein B mRNA editor associated with C-to-U RNA editing, had I-to-I RNA editing level decreased with its gene expression significantly increased. Such a finding might indicate that apart from A-to-I RNA editing, C-to-U RNA editing was also involved in ocular SARS-CoV-2 infections. Studies have shown that *APOBEC3C* is associated with infections of RNA viruses such as hepatitis and HIV [65, 66]. This finding thus implies the involvement of multiple RNA editing types during SARS-CoV-2 infection, which needs to be explored in further studies.

The study has observed dramatic changes in A-to-I RNA editing in ocular tissues upon SARS-CoV-2 infection. The biological impacts of such epigenetic changes on the eye remain largely undermined and are complex and context-dependent. The effects of such A-to-I RNA editing changes might be beneficial or adverse, depending on specific gene changes, which needs further investigation. Nevertheless, as ADARs and A-to-I RNA editing are considered important antiviral components in mammalian cells, such RNA editing response to SARS-CoV-2 infection is likely to contribute to the cellular antiviral process. For example, the *MAVS* gene showed the most evident changes in RNA editing across different tissues upon the SARS-CoV-2 infection. Interestingly, *MAVS* also showed significant expression changes. *MAVS* encodes a crucial intermediary protein in the virus-induced beta interferon signaling pathways and is essential for activating transcription factors that control the expression of beta interferon and play a role in the innate immune response against viruses [67–69]. Upon SARS-CoV-2 infection, *MAVS* could probably be activated and contribute to the production of interferons, which are key antiviral chemokines that limit viral replication and spread [70, 71]. However, abnormal A-to-I RNA editing has also been recently implicated in diseases, especially immune-related pathogenesis. Persistent SARS-CoV-2 infection might lead to over-activated, A-to-I RNA editing, which could be adverse to the eye. Further research is needed to understand further the consequences of A-I RNA editing in the eye and to determine whether it is ultimately beneficial or adverse.

While providing valuable insights into the potential role of A-to-I RNA editing in ocular manifestations during SARS-CoV-2 infection, our current study has some limitations. Firstly, our study was based on bioinformatics analysis of publicly available datasets, and further experimental validation is needed to confirm the findings. Further functional studies could be important for future perspectives to validate and investigate the functional consequences of the RNA editing changes identified in the current study, which could provide a deeper understanding of the underlying mechanisms. Secondly, the current study focused on A-to-I RNA editing, whereas other types of RNA editing, such as C-to-U editing, which may also play a role in ocular SARS-CoV-2 infections, as well as the interplay between these different types of RNA editing, need to be investigated in further study.

In conclusion, our findings revealed substantial changes in A-to-I RNA editing in ocular tissues upon SARS-CoV-2 infection and provided new insights into understanding ocular manifestations of COVID-19.

### Supplementary Information

**Supplementary Material 1.**

**Supplementary Material 2.**

**Supplementary Material 3.**

## Data Availability

The study includes original contributions that are downloaded from the European Nucleotide Archive (ENA) (https://www.ebi.ac.uk/ena), containing 37 samples of five ocular tissues from the PRJNA790648 [46], PRJNA688734 [47], and PRJNA731890 [48]. Any further inquiries or questions can be directed to the corresponding authors of the study.

## Abbreviations

A-to-I: Adenosine to inosine
COVID-19: Coronavirus disease 2019
ACE2: Angiotensin-converting enzyme 2
TMPRSS2: Transmembrane serine protease 2
ADARs: Adenosine deaminases that act on RNA
SNVs: Single nucleotide variations
GO: Gene Ontology
KEGG: Kyoko Encyclopedia of Genes and Genomes
GLM: General linear model
DRE: Differential RNA editing
FDR: False discovery rate
SIFT: Sorts intolerant from tolerant
