# Supplementary figures and images for: Ocular A-to-I RNA editing signatures associated with SARS-CoV-2 infection
Source: BMC Genomics. 2024 May 1;25:431. doi: 10.1186/s12864-024-10324-z (PMC11061923; doi:10.1186/s12864-024-10324-z)

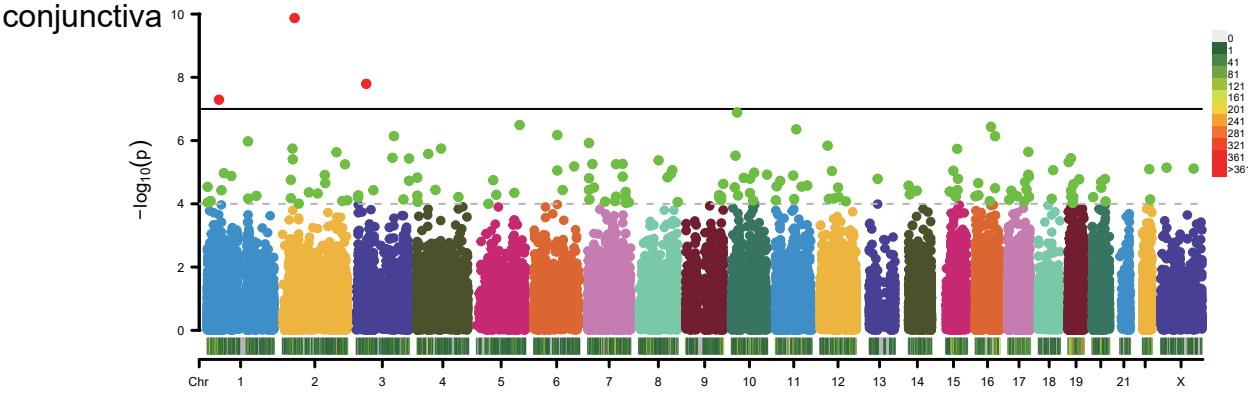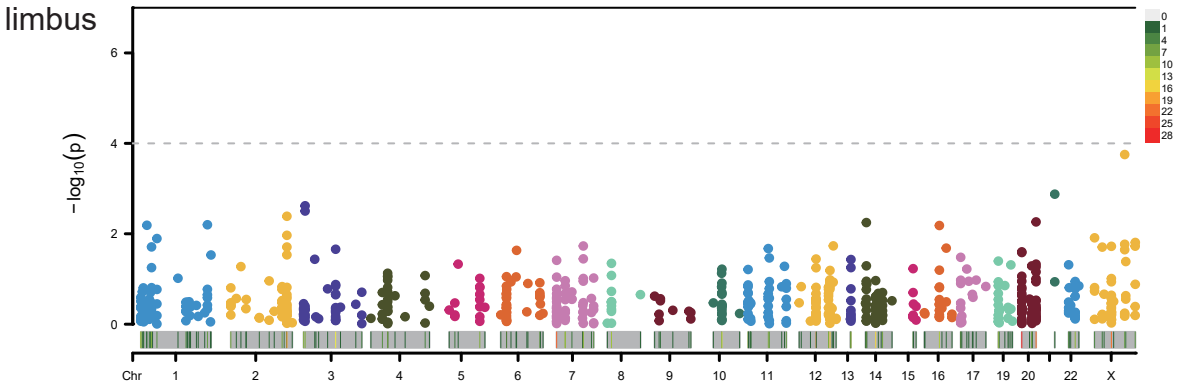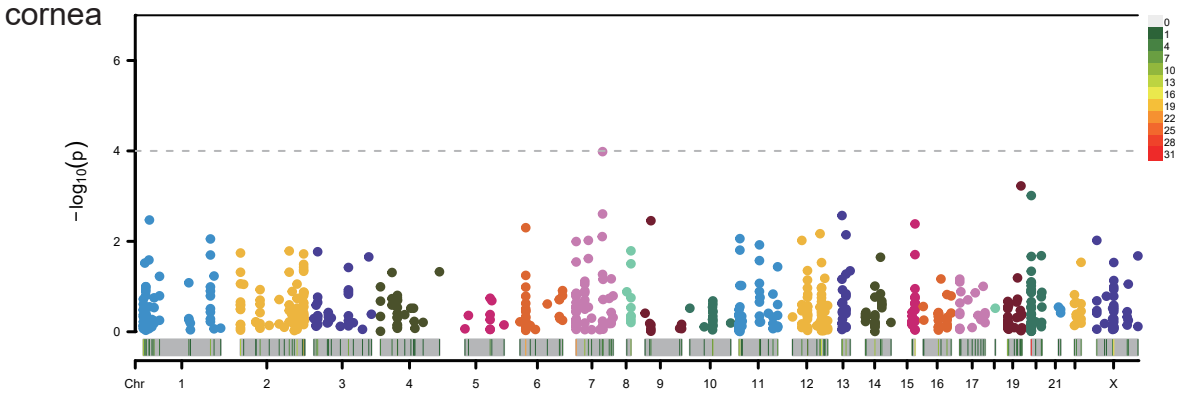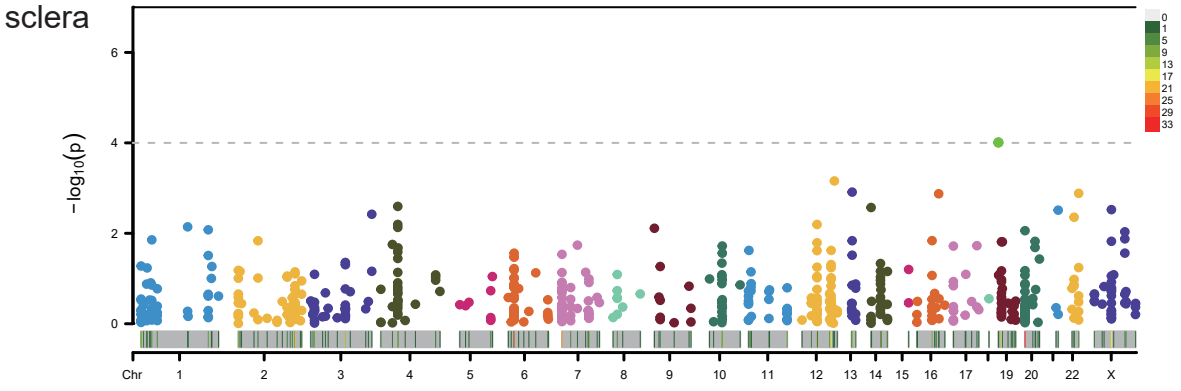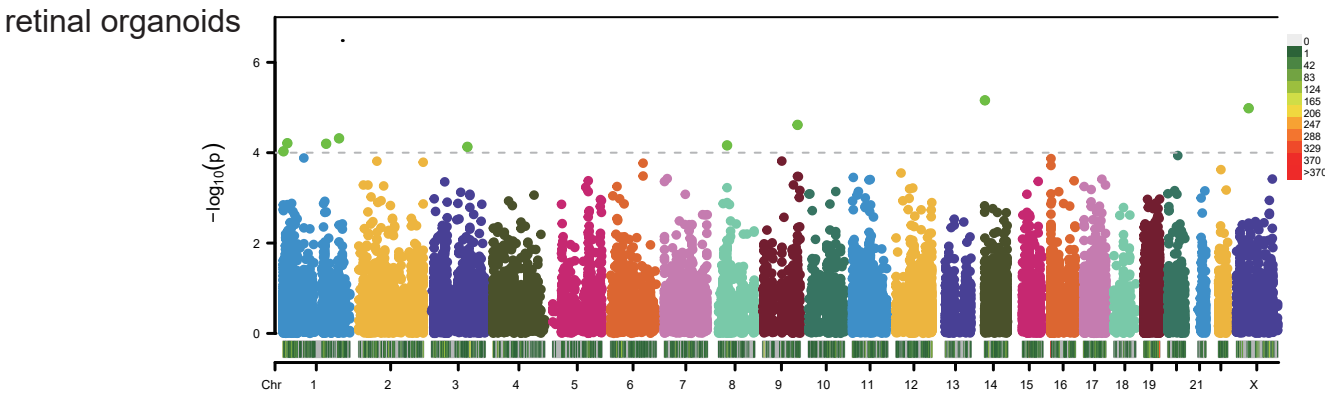

Supplement: Supplementary file 1 — Supplementary Material 1. [file 12864_2024_10324_MOESM1_ESM.pdf]

## conjunctiva

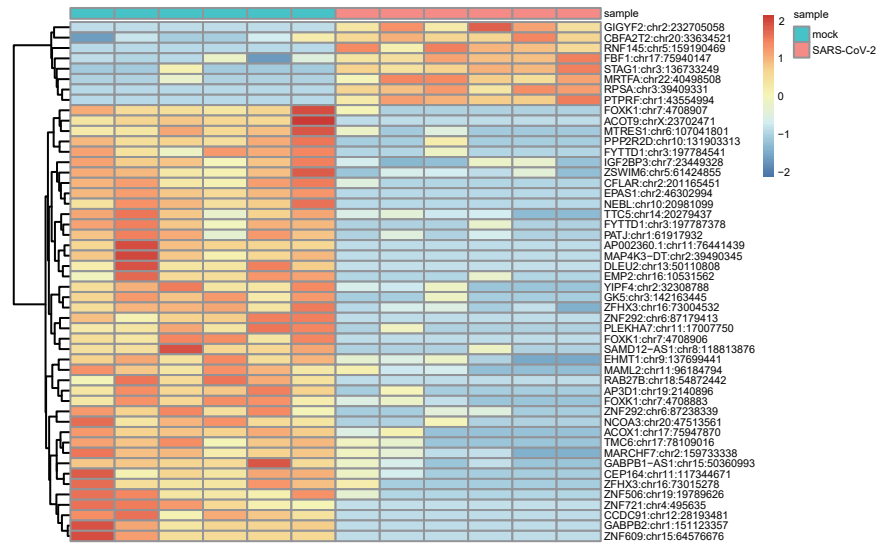

## limbus

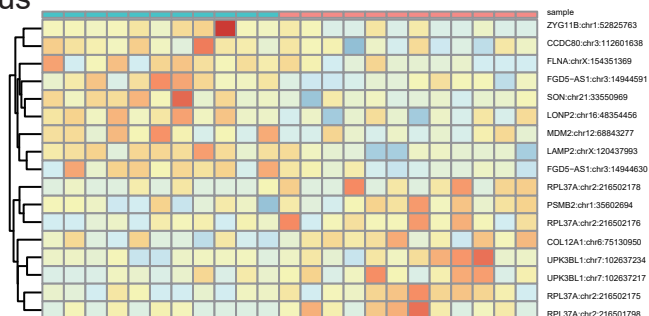

## cornea

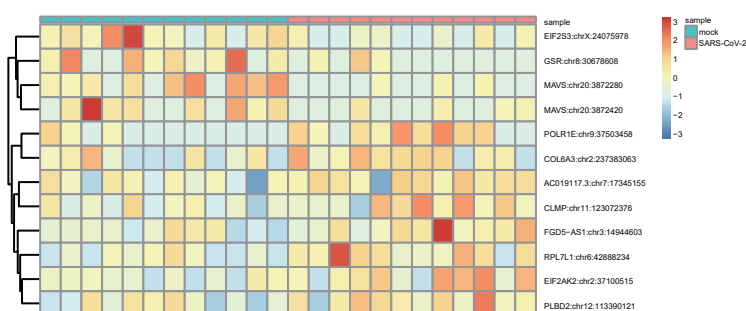

## sclera

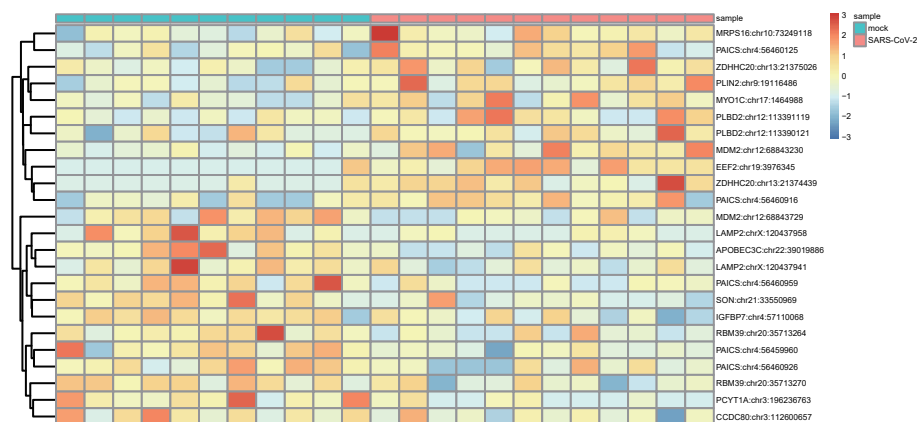

## retinal organoids

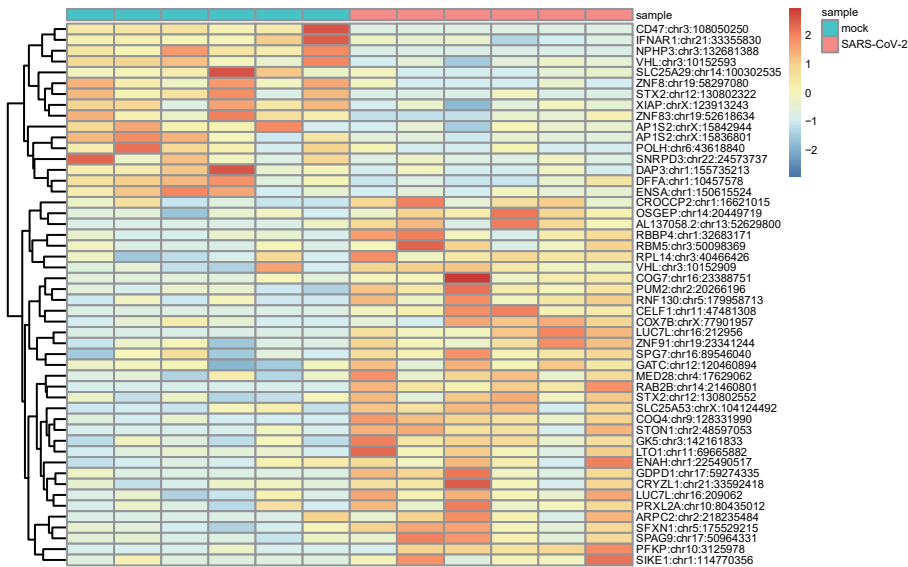

Supplement: Supplementary file 2 — Supplementary Material 2. [file 12864_2024_10324_MOESM2_ESM.pdf]

A

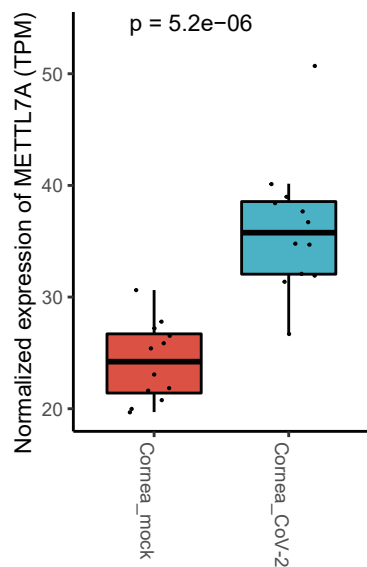

B

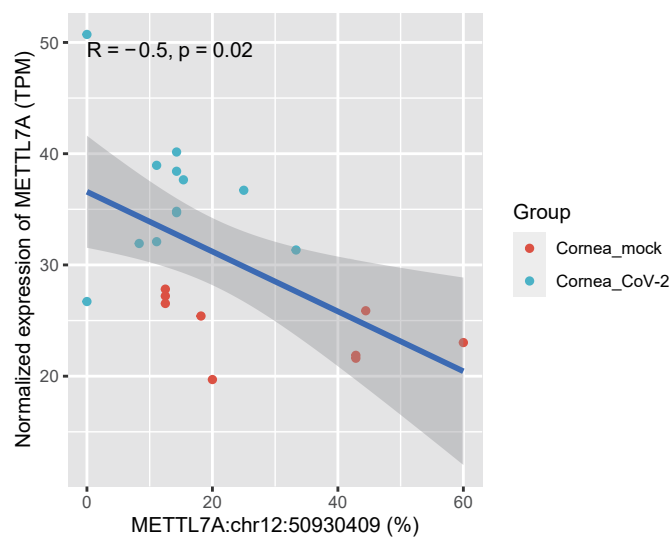

C

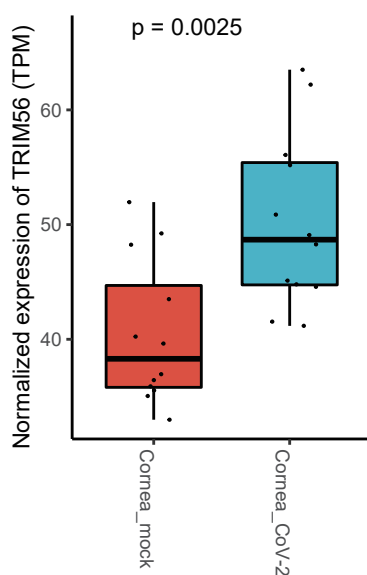

D

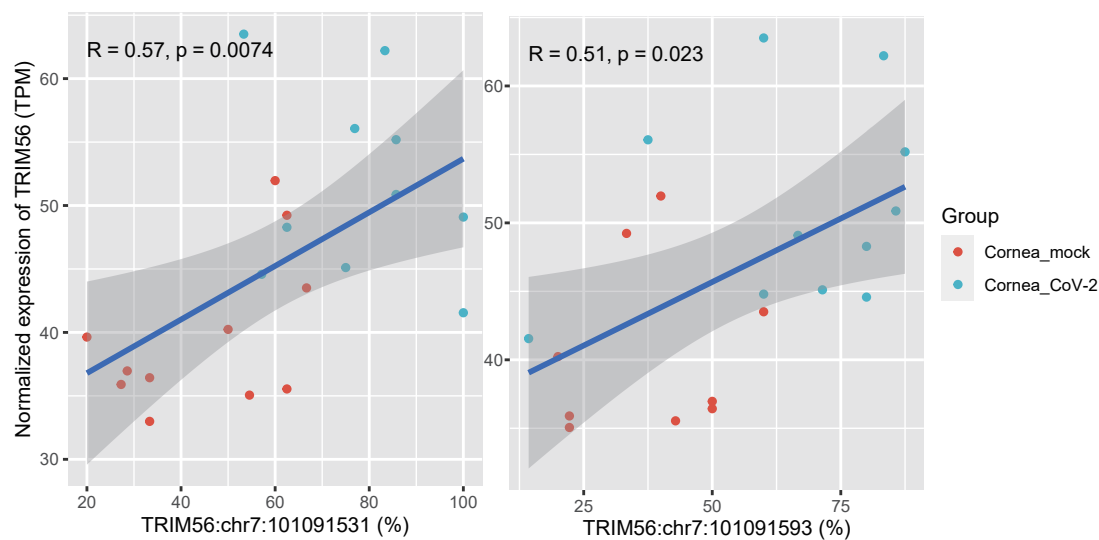

E

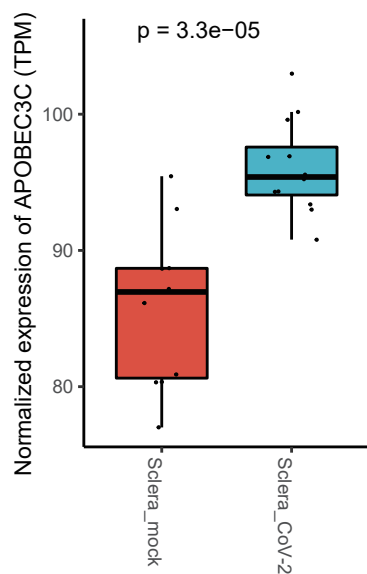

Supplement: Supplementary file 3 — Supplementary Material 3. [file 12864_2024_10324_MOESM3_ESM.pdf]
